# Proline and Proline Analogues Improve Development of Mouse Preimplantation Embryos by Protecting Them against Oxidative Stress

**DOI:** 10.3390/cells12222640

**Published:** 2023-11-16

**Authors:** Madeleine L. M. Hardy, Dheerja Lakhiani, Michael B. Morris, Margot L. Day

**Affiliations:** School of Medical Sciences, Faculty of Medicine and Health, The University of Sydney, Sydney, NSW 2006, Australia; madeleine.hardy@sydney.edu.au (M.L.M.H.); dlak8214@uni.sydney.edu.au (D.L.); m.morris@sydney.edu.au (M.B.M.)

**Keywords:** mitochondrial activity, oxidative stress, pipecolic acid, pre-implantation embryo development, proline, reactive oxygen species

## Abstract

The culture of embryos in the non-essential amino acid L-proline (Pro) or its analogues pipecolic acid (PA) and L-4-thiazolidine carboxylic acid (L4T) improves embryo development, increasing the percentage that develop to the blastocyst stage and hatch. Staining of 2-cell and 4-cell embryos with tetramethylrhodamine methyl ester and 2′,7′-dichlorofluorescein diacetate showed that the culture of embryos in the presence of Pro, or either of these analogues, reduced mitochondrial activity and reactive oxygen species (ROS), respectively, indicating potential mechanisms by which embryo development is improved. Inhibition of the Pro metabolism enzyme, proline oxidase, by tetrahydro-2-furoic-acid prevented these reductions and concomitantly prevented the improved development. The ways in which Pro, PA and L4T reduce mitochondrial activity and ROS appear to differ, despite their structural similarity. Specifically, the results are consistent with Pro reducing ROS by reducing mitochondrial activity while PA and L4T may be acting as ROS scavengers. All three may work to reduce ROS by contributing to the GSH pool. Overall, our results indicate that reduction in mitochondrial activity and oxidative stress are potential mechanisms by which Pro and its analogues act to improve pre-implantation embryo development.

## 1. Introduction

The development of preimplantation embryos in vitro is associated with higher levels of cellular stress than those developed in vivo [1]. In vitro developed embryos are slower to reach each stage and are generally of lower quality than their in vivo developed counterparts [2]. In vitro embryo culture disrupts normal pathway activities, including those for metabolism and mTOR signalling [3,4]. mTOR is at the centre of intracellular signalling, converting cues obtained from the extracellular environment, including amino acids, into cellular responses such as cell growth and proliferation [4]. As the extracellular environment differs between embryos developed in reproductive fluids in vivo compared to embryos cultured in vitro in media, a major focus has been on creating a culture environment that better recapitulates the success of embryo development in vivo [5,6,7]. In particular, despite substantial improvements to media, cultured embryos are under greater oxidative stress and contain significantly higher amounts of reactive oxygen species (ROS) than those developed in vivo, in part due to the lack of antioxidants in culture media [8,9,10].

ROS are unstable, highly reactive intermediates derived from molecular oxygen from various cellular processes [11], principally the electron transport chain (ETC) [12] and the action of the NOX family of NADPH oxidases [13,14]. The principal species are superoxide anions (O_2_^•–^), hydrogen peroxide (H_2_O_2_) and hydroxyl radicals (·OH). A small percentage of electrons leaking from the ETC produce superoxide [15], which is very rapidly converted to H_2_O_2_ by superoxide dismutase (SOD) [16]. Excess production of ROS in a cell results in an imbalance in the redox state sufficient to result in oxidative stress [17]. The homeostatic range for O_2_^•–^ and H_2_O_2_ depend on the cell type and conditions, but are ~200 pM and 1–10 nM, respectively [18,19]. In this range, they are important contributors to cell signalling [10], helping to regulate the cell cycle, cell proliferation and apoptosis [20,21], thereby positively influencing embryo viability. Excess production, however, compromises a range of cellular functions, which can lead to cell death [22,23].

Not surprisingly, then, mitochondrial activity, and metabolic activity in general, have been linked to embryo viability. The ‘goldilocks principle’ states there is an optimal range of metabolic activity and, for example, an embryo with pyruvate consumption in the range 4–10 pmol/h has improved viability compared to those with a rate of <4 and >10 pmol/h [24]. Often these hormetic ranges are incompletely defined. For example, embryos which have lower amino acid (AA) turnover are more viable than embryos that have a higher AA turnover, with embryos that arrest during development having an AA turnover 3.7-fold higher than those that develop to the blastocyst stage [25,26,27,28]. However, the lower boundary of turnover required to support good viability is not well defined. Similarly, mitochondrial activity at the lower end of the spectrum is beneficial for development due to reduced mitochondrial ROS production (and therefore reduced oxidative stress) [29,30,31], but the lower end of this range is, again, poorly defined.

Consistent with this, human oocytes exposed to follicular fluid with excess ROS have reduced fertilisation and produce lower quality embryos [10]. Oocytes and embryos cultured in antioxidants, such as melatonin [32,33], glutathione (GSH) [34], or ascorbic acid [35], have reduced DNA fragmentation [36] and improved developmental progression and subsequent implantation success.

Some groups of AAs added to culture medium also improve embryo development [37]. In addition, L-proline (Pro) or L-glutamine (Gln) added individually achieve this [38], whereas a range of other AAs and AA analogues fail to do so, or prevent the actions of Pro and L-glutamine, or impair development [4,38,39,40]. The advantage of adding AAs individually is that understanding the molecular mechanisms underlying beneficial effects is easier. In this study, we extend a range of previous work on the molecular mechanisms of action of Pro driving development [4,38,39,41] by looking at the effect of it, as well as three Pro analogues, on mitochondrial activity and ROS levels at pre-implantation stages.

Pro is a conditional non-essential AA whose concentration in follicular and oviductal fluid across a number of mammalian species ranges from 0.05–0.6 mM [42,43,44,45]. AA transporters for Pro are present throughout development of the pre-implantation embryo [40,46,47,48,49,50,51,52]. When 400 µM Pro is added to culture medium, it is taken up into the embryo to millimolar concentrations [46] and improves in vitro embryo development in a growth factor-like manner, from the late 2- to 8-cell stage out to the blastocyst stage, including hatching [38]. This exogenous concentration is consistent with that in reproductive fluids [42,43,44,45], the *K_m_* of various Pro transporters [47,53], and its efficacy in a variety of in vivo and in vitro development and non-development models [4,38,39,54,55,56].

The Pro-related mechanisms at play in the preimplantation embryo are not fully understood but rely, at least in part, on mTOR pathway activity [38]. However, since all Pro transporters have multiple substrates, its uptake can be sufficiently limited by competitive inhibitors to prevent its beneficial effects [38,39,57]. Thus, the use of groups of amino acids, which do not take competitive inhibition into consideration, can fail to achieve the desired effects.

Pro is metabolised in the Pro cycle [58] by proline oxidase (POX), an inner mitochondrial membrane enzyme, to form pyrroline-5-carboxylate (P5C) [59]. This conversion produces a burst of ROS (O_2_^•–^) within mitochondria via the ETC when cells are exposed to Pro, and accounts for a small portion of cellular ROS production [60]. However, chronic exposure to Pro (hours to days) can reduce cellular ROS levels by a variety of context-dependent mechanisms, including Pro (i) acting as a ROS scavenger through its secondary amine group [61,62,63], (ii) contributing to the production of the cell’s major antioxidant, GSH [63] and (iii) reducing mitochondrial activity by downregulating the expression of ETC genes [13]. Thus, ROS reduction is a potential mechanism by which Pro improves embryo development. Consistent with this, in vitro fertilisation of mouse oocytes in the presence of Pro for several hours lowers levels of ROS and improves subsequent development to the blastocyst stage [39]. This reduction in ROS by Pro does not occur with other AAs, such as glycine, betaine and histidine [39], and this selectivity for Pro occurs in a variety of other cellular systems [54,56,57,60,64].

**Figure 1:**
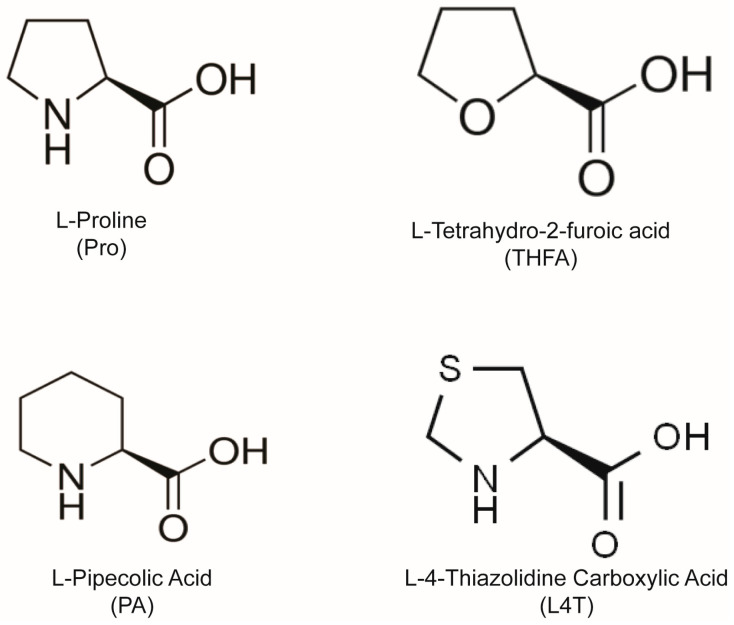
Chemical structures of Pro and Pro analogues, L-tetrahydro-2-furoic acid (THFA), L-pipecolic acid (PA) and L-4-Thiazolidine carboxylic acid (L4T).

Pro analogues, such as L-pipecolic acid (PA) and L-4-thiazolidine carboxylic acid (L4T, also referred to as thioproline), also contain secondary amines (Figure 1), and can reduce oxidative stress in other cells, including oocytes [39,63,65]. In this study, we show that Pro and these Pro analogues reduce oxidative stress and mitochondrial activity in 2-cell and 4-cell embryos, as well as improve embryo development to the blastocyst stage. The results are consistent with Pro reducing ROS by reducing mitochondrial activity while PA and L4T may be acting as ROS scavengers. All three may work to reduce ROS by contributing to the GSH pool.

## 2. Materials and Methods

### 2.1. Animals (Mus Musculus)

Outbred Quackenbush Swiss (QS) mice (Animal Research Centre, Perth Australia and Lab Animal Services, The University of Sydney) were used in accordance with the Australian Code of Practice for the Care and Use of Animals for Scientific Purposes and was approved by the University of Sydney Animal Ethics Committee as per the requirements of the NSW Research Act (approval numbers 824—June 2015–June 2019, 1569—July 2019–July 2021, 1877—March 2021–March 2024). Mice were housed under a 12 h light, 12 h dark cycle.

### 2.2. Zygote Collection and Media Preparation

In this study, 3–5-week-old female mice were superovulated by intraperitoneal injection with 10 IU pregnant mares’ serum (PMS) (Intervet, VIC, Australia), followed 48 h later by an injection of 10 IU human chorionic gonadotropin (hCG) (Intervet, VIC, Australia). The females were then paired immediately overnight with 2–8-month-old males. Mating was confirmed the following day by the presence of a vaginal plug and the females euthanised by cervical dislocation 22–24 h post hCG and the oviducts removed. Cumulus masses containing zygotes were dissected from the oviducts in HEPES-buffered modified human tubal fluid (HEPES-ModHTF) [38] and treated with hyaluronidase (1 mg/mL in HEPES-ModHTF) for 5 min at room temperature to remove the cumulus cells. Zygotes were then transferred to modified human tubal fluid (ModHTF) without Gln. Both media were supplemented with 0.3 mg/mL bovine serum albumin (BSA), buffered to pH 7.4 and adjusted to 270 mOsm/kg.

### 2.3. Embryo Culture and Scoring Developmental Progression

Embryos were placed individually in 100 µL ModHTF in wells of round-bottomed 96-well plates (Corning) overlaid with mineral oil either in the absence of AAs (no AA) or in the presence of 400 µM of the following: Pro, PA, THFA, L4T, or THFA + Pro. Embryos were cultured in a humidified incubator at 37 °C and 5% CO_2_, and development was scored at 24 h for 2-cell development, 48 h for ≥5-cells, 60 h for ≥8-cells, 72 h for ≥morula, 96 h for blastocysts, and 120 h for hatched blastocysts. Embryo morphology was examined each day, and only morphologically normal embryos were included in this study.

### 2.4. Imaging of Reactive Oxygen Species and Mitochondrial Activity

Embryos were cultured to the 2- or 4-cell stage in ModHTF medium containing no AAs or containing 400 µM of the following: Pro, PA, THFA, L4T, or THFA + Pro. Embryos were then stained for 30 min at 37 °C and 5% CO_2_ with a combination of 100 nM tetramethylrhodamine methyl ester (TMRM) and 10 µM 2′,7′-dichlorofluorescein diacetate (DCFDA). TMRM accumulates in mitochondria, and its fluorescence correlates with mitochondrial hyperpolarisation [66], and hence mitochondrial activity, while H_2_-DCFDA detects ROS [67]. Both dyes were prepared in ModHTF medium containing no AAs or the treatments, as above. Embryos were then washed twice in ModHTF containing the treatments to remove excess stain. A 35 mm glass-bottomed petri dish was prepared containing 10 μL drops of ModHTF which contained each of the treatments under oil. The mitochondrial uncoupler, carbonyl cyanide-4 (trifluoromethoxy) phenylhydrazone (FCCP; 5 µM), was used as a positive control for mitochondrial activity, and H_2_O_2_ (60 µM) as a positive control for ROS. Embryos were imaged using a LSM800 confocal microscope (Carl Zeiss, Jena, Germany) with the incubation chamber set to 37 °C and 5% CO_2_ for live-cell imaging. Embryos were located, and the focal plane was set through the nuclei for each blastomere. Microscope settings were reused between each condition and for each experiment. A 20× objective, 524 nm and 488 nm lasers, and Zen 2.6 Blue Edition software (Carl Zeiss) were used.

### 2.5. Analysis of Confocal Images

Images were analysed using Fiji by Image J to obtain the integrated density of fluorescence for TMRM and H_2_-DCFDA.The integrated density of individual blastomeres was measured in three equidistant locations around the nuclei using an ellipse of equal size. The corrected total cell fluorescence was then calculated using the following formula [68]:

Corrected total cell fluorescence = integrated density − (background fluorescence × ellipse area)

### 2.6. Measurement of L-[^3^H]-Pro Uptake in 2- and 4-Cell Embryos

Embryos were isolated as zygotes and cultured to the 2- or 4-cell stage in ModHTF containing no AAs (as above). Embryos were then incubated in 20 µL assay medium consisting of 1 µM L-[^3^H]-Pro (1 mCi/mL L-[2,3,4,5-^3^H]-proline; Perkin-Elmer, Glen Waverly, VIC, Australia, #NET483001MC) in HEPES-ModHTF ± unlabelled 400 µM Pro or THFA. In groups of four, embryos were incubated for 100 min at 37 °C in each treatment, then washed through at least 8 wash drops of cold (4 °C) HEPES-ModHTF and placed onto a 96-gridded filter mat (Perkin-Elmer, #1450-421). The mat was placed inside a plastic sleeve, 4 mL ULTIMA GOLD scintillation fluid (Perkin-Elmer, Australia, #6013371) was added, and the sleeve heat sealed. Samples were counted for 30 min using a MicroBeta TriLux Plate Counter (Perkin-Elmer, Australia). The rate of L-[^3^H]-Pro uptake (fmol min^−1^ embryo^−1^) was determined using a standard curve created for each experiment from standards serially diluted from a stock of 1 µM L-[^3^H]-Pro in HEPES-ModHTF.

## 3. Results

### 3.1. Pro, PA and L4T Improve Embryo Development to the Blastocyst Stage

**Figure 2:**
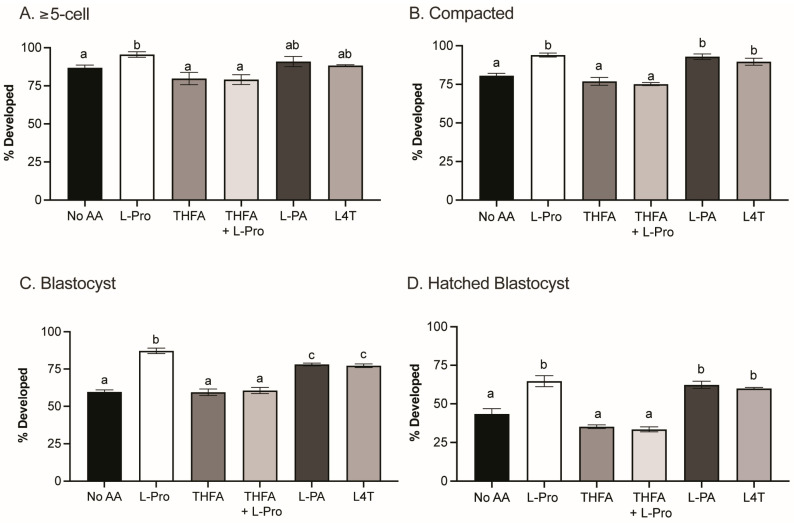
Preimplantation embryo development in vitro in the presence and absence of Pro and Pro analogues. Zygotes were cultured ±400 µM of Pro, THFA, THFA + Pro, PA, or L4T for 5 days with development scored daily. Graphs show development to the (**A**) ≥5-cell, (**B**) compacted, (**C**) blastocyst and (**D**) hatched blastocyst stages. Bars represent mean ± SEM, obtained from a minimum of three independent experiments containing at least 12 embryos per condition per experiment. Data were compared by one-way ANOVA with a Tukey’s multiple comparisons test. Bars with a different letter are significantly different (*p* < 0.05).

The presence of Pro in embryo culture medium increases the percentage of embryos that develop to the blastocyst stage [38]. In order to determine how Pro mediates this improvement and whether Pro analogues have a similar effect, zygotes were cultured in medium containing no AAs or 400 µM of Pro, THFA, Pro + THFA, PA or L4T. None of the treatments had any effect on cleavage to the 2-cell stage (*p* > 0.05). Pro improved embryo development from the ≥5-cell stage onwards (Figure 2A,B) and increased the percentage of embryos that developed to the blastocyst stage (Figure 2C) and hatched (Figure 2D), consistent with previous findings [38]. The Pro analogues, PA and L4T, also improved development at each of these stages (Figure 2), although the increase in development to the blastocyst stage was not as great as that with Pro (Figure 2C). In contrast, the Pro analogue THFA, which inhibits the metabolism of Pro to P5C by POX [60], prevented the Pro-mediated improvement but had no effect on development alone (Figure 2A–D).

### 3.2. THFA Does Not Prevent the Uptake of Pro in 2- or 4-Cell Embryos

**Figure 3:**
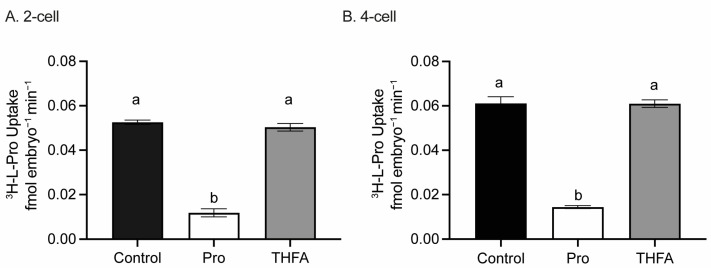
Rate of uptake of radiolabelled Pro in the presence of excess unlabelled Pro or THFA. Embryos were incubated in 1 μM L-[^3^H]-Pro in the absence (control) or presence of 5 mM Pro or 400 μM THFA for 100 min. Uptake at the (**A**) 2-cell and (**B**) 4-cell stages was quantified using scintillation counting. Data represent the mean rate of uptake ± SEM from three independent experiments with 12 embryos per treatment performed in triplicate in each experiment. Data were analysed by one-way ANOVA with Tukey’s multiple comparisons test. Bars with a different letter are significantly different (*p <* 0.05).

**Figure 4:**
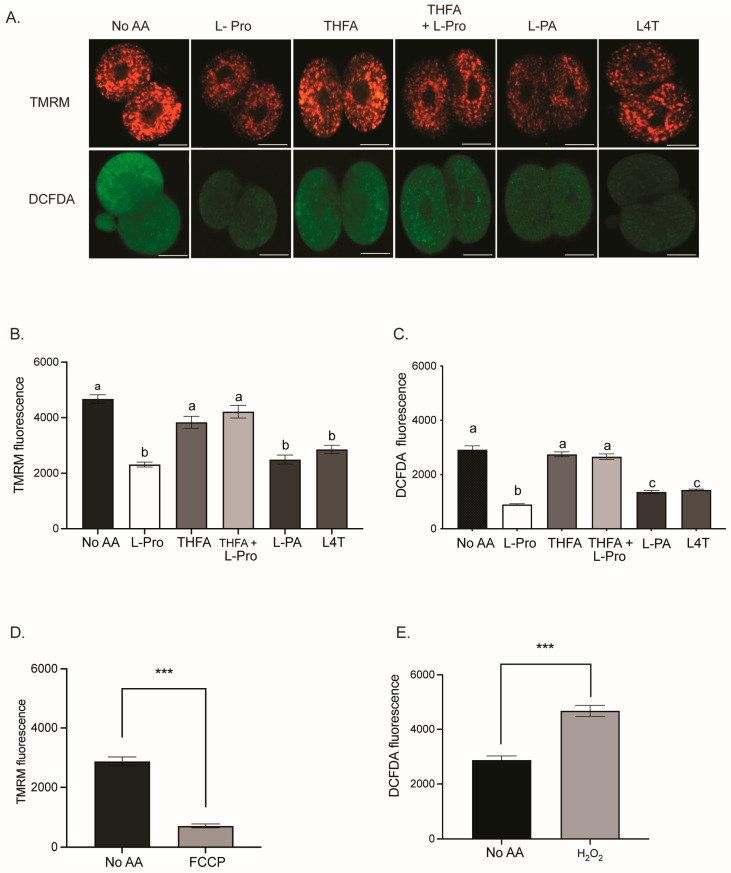
The effect of Pro and Pro analogues on the mitochondrial activity and ROS content in 2-cell embryos. Zygotes were cultured in ModHTF in the presence or absence of 400 µM Pro, THFA, THFA+ Pro, PA, or L4T for 24 h to the 2-cell stage. Embryos were loaded with 10 µM DCFDA for ROS and 100 nM TMRM for mitochondrial activity and imaged using confocal microscopy. (**A**) Representative image of embryos in each condition. Scale bar = 20 µm. Cell fluorescence for (**B**) TMRM and (**C**) DCFDA. Control embryos were stained with DCFDA and TMRM and transferred to a 10 µL drop of medium containing (**D**) 5 µM carbonyl cyanide-p-trifluoromethoxyphenylhydrazone (FCCP) or (**E**) 60 µM H_2_O_2_ prior to imaging. Bars represent mean ± SEM of 25–78 embryos obtained from at least three independent experiments. Data were analysed using a one-way ANOVA with a Tukey’s multiple comparisons test. Bars with different letters are significantly different (*p* < 0.05) and *** represents (*p* < 0.001).

Since THFA has a structure similar to Pro (Figure 1), it may have reduced the beneficial effect of Pro on development by competing for transporter-mediated uptake of Pro into the embryo. However, THFA did not affect uptake of L-[^3^H]-Pro in 2- (Figure 3A) or 4-cell stages (Figure 3B). The addition of excess unlabelled Pro (5 mM) greatly reduced uptake of radiolabelled Pro demonstrating uptake was via a saturable transporter (Figure 3).

### 3.3. Pro and Analogues PA and L4T Decrease Mitochondrial Activity and ROS in 2- and 4-Cell Embryos

**Figure 5:**
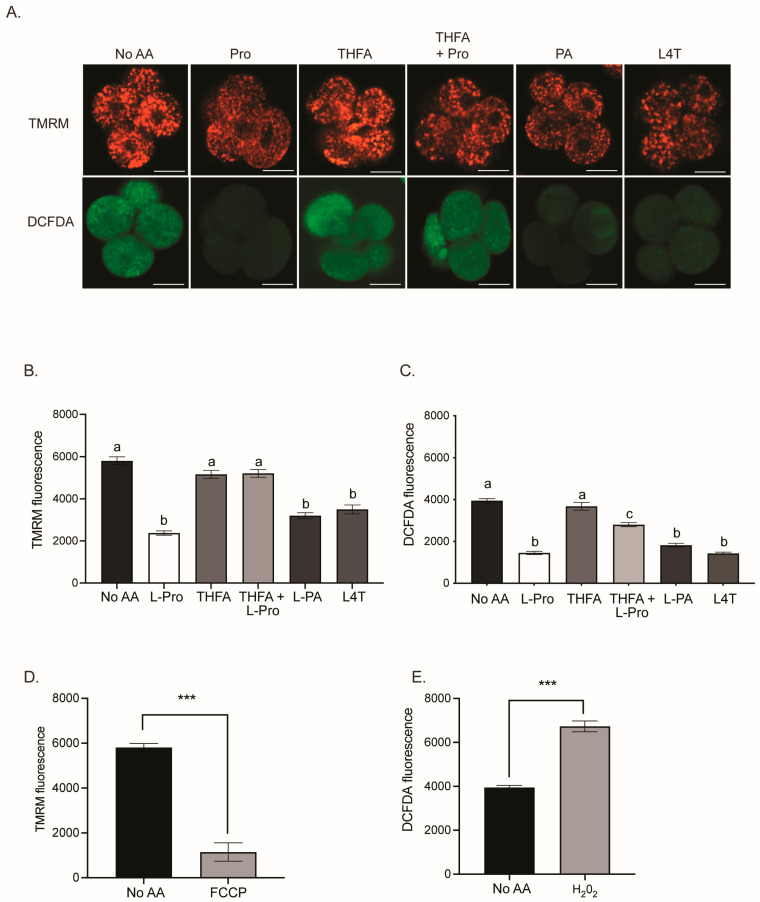
The effect of Pro and Pro analogues on the mitochondrial activity and ROS content in 4-cell embryos. Zygotes were cultured in ModHTF in the presence or absence of 400 µM Pro, THFA, THFA+ Pro, PA, or L4T for 48 h to the 4-cell stage. Embryos were loaded with 10 µM DCFDA for ROS and 100 nM TMRM for mitochondrial activity and imaged using confocal microscopy. (**A**) Representative image of embryos in each condition. Scale bar = 20 µm. Cell fluorescence for (**B**) TMRM and (**C**) DCFDA. Control embryos were stained with DCFDA and TMRM and transferred to a 10 µL drop of medium containing (**D**) 5 µM carbonyl cyanide-p-trifluoromethoxyphenylhydrazone (FCCP) or (**E**) 60 µM H_2_O_2_ before imaging. Bars represent mean ± SEM of 29–65 embryos obtained from at least three independent experiments. Data were analysed using a one-way ANOVA with a Tukey’s multiple comparisons test. Bars not sharing the same letter are significantly different (*p* < 0.05) and *** represents (*p* < 0.001).

The mechanism underpinning the improvement in development mediated by Pro, PA and L4T is unknown. Furthermore, embryos are sensitive to oxidative stress [8,9], and the quiet embryo hypothesis suggests that embryos with lower metabolism, and hence lower mitochondrial activity, are more viable [26]. Thus, the effect of Pro and its analogues on ROS levels and mitochondrial activity was investigated. Embryos were cultured from the zygote to the 2- or 4-cell stage in medium containing either no AAs or 400 µM of Pro, THFA, Pro + THFA, PA or L4T. Embryos were loaded with DCFDA, to measure the amount of ROS, and TMRM, to measure mitochondrial activity, and imaged by confocal microscopy. Pro, L4T and PA all decreased mitochondrial activity and ROS levels in 2- and 4-cell embryos compared to no AA (Figure 4 and Figure 5). THFA alone did not affect mitochondrial activity or ROS, while THFA prevented the decrease in mitochondrial activity and ROS induced by Pro (Figure 4 and Figure 5). When embryos were cultured in the presence of the mitochondrial uncoupler, FCCP, there was a reduction in TMRM fluorescence, as expected (Figure 4D and Figure 5D). H_2_O_2_, a potent inducer of ROS, was used as a positive control for DCFDA and caused an increase in ROS, as expected (Figure 4E and Figure 5E).

## 4. Discussion

Pro improves development from the late 2-cell to 8-cell stages, and this advantage is maintained out to the hatching blastocyst stage (Figure 2) [38]. In this study, we show that during this critical period, Pro, PA and L4T all reduce mitochondrial activity and oxidative stress in 2- and 4-cell embryos (Figure 4 and Figure 5).

The results are consistent with the ‘quiet’ embryo hypothesis in which improved development is coupled to decreased metabolic processes, such as glycolysis, oxidative phosphorylation and AA turnover [3], which in turn reduce ROS [27,28,69]. On the other hand, embryos in which these processes are upregulated undergo developmental arrest more frequently [27]. We have also shown that Pro and PA, added only during fertilisation, result in ‘quiet’ oocytes with reduced mitochondrial activity and ROS, which then have improved development from compaction onwards [39]. Pro and PA also prevent ROS-mediated damage to boar sperm [65], including rescue of H_2_O_2_-induced motility loss. More generally, in other cell types, Pro, PA and L4T maintain cell survival after exposure to ROS, such as H_2_O_2_ [63].

Pro-mediated improvement in development and differentiation first depends on its uptake into cells via AA transporters, and competitive inhibitors of its uptake prevent its effects [38,39,50,57]. In the embryo, the competitive inhibition profile indicates B^0^AT1 is the responsible transporter [38]. During this critical period of late 2- to 8-cell stage, other transporters are also expressed in the mouse embryo, including GLYT1, which may also contribute to Pro uptake [70]. Although not extensively studied, a number of transporters also take up PA and L4T during or before this time. For example, PA can be transported by SIT1, PROT and GLYT1 [39,40,46,47,71,72]. Less is known about transporters of L4T, but it can be transported by PAT2 during this time [73,74].

Once inside the embryo, Pro, PA and L4T can reduce ROS through a variety of mechanisms. Below, we outline mechanisms consistent with our data and note that each of these molecules probably act via multiple mechanisms.

### 4.1. Reduction in Mitochondrial Activity Reduces ROS

Since mitochondria are a major source of ROS generation [75,76], suppression of mitochondrial activity per se would be expected to reduce ROS. The reduction of both mitochondrial activity and ROS by Pro has been seen in other cells, including vitrified oocytes [77], but is not fully understood [78]. In fact, the result is counter-intuitive: (a) Pro can be converted to P5C (by POX) and then to α-ketoglutarate (via glutamate), and, hence, enter the TCA cycle [59,79], which ultimately boosts ETC activity. (b) POX is coupled to succinate dehydrogenase of Complex II in the ETC [13], and the high-energy electrons generated by its catalysis of Pro can be donated to the ETC to promote ATP production [80]. (c) A by-product of the oxidation of Pro by POX is the generation of O_2_^•–^ [81], and forced expression of POX in human colon cancer DLD-1 cells results in an acute burst of ROS [13]. Thus, each of these mechanisms would be expected to increase mitochondrial activity and ROS, yet the opposite is occurring here in embryos.

However, whilst forced expression of POX results in an acute burst of ROS [13], the long-term effect over several days is reduced mitochondrial activity and ROS [13]. POX is directly coupled to ETC Complex II, and its forced overexpression ultimately results in a decrease in expression of a range of ETC complex proteins; i.e., mitochondrial activity goes down over time, and ROS with it [13]. Furthermore, forced overexpression of POX coupled with prolonged exposure to added Pro exacerbates the downregulation of mitochondrial activity and ROS [13], presumably because this further upregulates the expression of *Prodh*, the gene for POX. Consistent with this, exposure of the porcine trophectoderm cell line, pTr2, to 0.5 mM Pro eventually results in a 3-fold increase in POX protein [82].

In our study, we have an analogous situation: Zygotes have been exposed long-term to added Pro (24 h to reach the 2-cell stage and 48 h for the 4-cell stage), after which time mitochondrial activity and ROS are reduced compared to embryos never exposed to Pro. Accompanying this, gene expression of *Prodh* in these Pro-treated 2-cell embryos increases to that of highly expressed 18S ribosomal RNA (C_t_ = 12.5 and 13.5, respectively), whilst expression of *Prodh* in the untreated controls is undetectable (as measured by TaqMan PCR). Therefore, the mechanisms at play in terms of reduced mitochondrial activity and ROS that we are observing in early embryos may be similar to DLD-1 cells overexpressing POX ± Pro [13].

Neither PA nor L4T is likely to act in this fashion. PA is metabolised by pipecolic acid oxidase (PIPOX) [83], and while it can be found in mitochondria, in most cell types it is predominantly located in the peroxisome [84,85]. Furthermore, PIPOX is not directly coupled to the ETC, but it does produce H_2_O_2_ during catalysis [85]. In addition, while PIPOX can catalyse both PA and Pro [86], PA is not a substrate for POX [87,88]. On this basis, PA is unlikely to suppress mitochondrial activity and ROS through effects on ETC activity. Similarly, L4T is unlikely to be a substrate for POX [87,88], although the enzymes which metabolise it and their locations are poorly characterised [89]. In summary, we propose that PA and L4T reduce ROS by different mechanisms (see below), but it is not clear how they reduce mitochondrial activity.

### 4.2. Direct Scavenging of ROS

Pro is the only common AA with a secondary amine [90]. This lowers the ionisation potential compared to the primary amine in the other proteinogenic AAs [61,63] and allows Pro to reduce ROS levels by reacting with and stabilising free radicals, such as •OH and singlet oxygen [61,63]. Furthermore, the addition of Pro (0.4–1 mM) to zygotes for ≥24 h results in its substantial intracellular accumulation (7–35 mM) even in isosmotic medium [47]. However, results with the POX inhibitor, THFA (Figure 2, Figure 3, Figure 4 and Figure 5), indicate Pro is not acting as a ROS scavenger in the early embryo. THFA reversed the Pro-mediated improvement in embryo development (Figure 2) and the reduction in ROS (Figure 4C and Figure 5C), yet because it prevents the conversion of Pro to P5C [55,60] its presence would be expected to further increase the intracellular concentration of Pro and hence its scavenging capacity. THFA did not prevent Pro uptake (Figure 3) nor on its own change embryo development or reduce ROS (Figure 2, Figure 4 and Figure 5). These results are consistent with its lack of a secondary amine group (Figure 1) and with results showing that Pro, but not THFA, rescues boar sperm from oxidative stress induced by H_2_O_2_ [65]. In summary, these data indicate that Pro-mediated reduction in ROS arises downstream of intact Pro; e.g., by POX-mediated reduction in mitochondrial activity as described above.

On the other hand, PA and L4T may be acting as ROS scavengers. The secondary amine in the six-membered ring of PA is expected to have an even lower ionisation potential than Pro and therefore prove to be a better ROS scavenger than Pro itself [61,63]. Similarly, L4T can function as a sulfhydryl-mediated antioxidant [91,92].

### 4.3. Reduction of ROS through Production of GSH

In other cell types, up to 75% of Pro is converted to glutamate [93], after which, instead of being converted to α-ketoglutarate, it can be used to produce the potent antioxidant, GSH [94]. The production of GSH from Pro may partly explain the decrease in ROS seen in embryos (Figure 4 and Figure 5). Pro-mediated increase in GSH also occurs in boar sperm [65] and HEK293 cells [63], and it reduces ROS and improves maturation of porcine oocytes [95].

Glutamate may exist in preimplantation embryos in separate pools, which serve different functions. This is analogous to embryonic stem cells where a separate pool of glutamate is reserved for the activation of metabotropic glutamate receptor 5 (GMR5) for maintenance of pluripotency. Thus, it is possible that there is a pool of glutamate reserved for the formation of GSH [96].

Metabolism of PA can also result in GSH production since PIPOX converts PA to the precursor P6C [85,97,98]. L4T is metabolised through a series of enzymatic steps to form cysteine [89,99], the rate-limiting component of GSH formation [100]. Addition of L4T to HeLa cells increases GSH and protects against oxidative stress [92].

Several other mechanisms also control Pro-mediated improvement in embryo development [38] and the differentiation of ES cells [41,50,57,101]. For example, Pro activates the mTOR, ERK and Akt pathways [4,38,41,50,57,102]. However, it is not known if PA and L4T also activate these pathways in embryos. PA does not stimulate differentiation of ES cells [57], possibly due to the absence of a transporter. The effect of L4T on ES cell has not been determined.

It is also not known at this stage if any of the Pro-mediated mechanisms that improve development at the late 2-cell to 8-cell stage are required to maintain that developmental advantage out to the hatching blastocyst stage. What does appear to be clear, however, is that Pro acts at various points in development from the oocyte to the production of neurectoderm, consistent with its growth factor-like properties [4,38,39,41,52].

## 5. Conclusions

In summary, the addition of Pro to embryo culture improves preimplantation embryo development and reduces ROS, suggesting that a reduction in ROS may contribute to this improvement in development. The Pro-mediated reduction in ROS is consistent with the following mechanisms: Reduction in mitochondrial activity via downregulation of the expression of ETC components [13] and increased GSH production [103]. For PA and L4T, the possible mechanisms would appear to be direct ROS scavenging and GSH production, although the reason for the concomitant reduction in mitochondrial activity remains unknown. In all cases, the ‘quieter’ metabolic status of these treated embryos is consistent with their improved capacity to develop to the blastocyst stage [3,39].

## Data Availability

The data underlying this article are available in the article.
